# Hypoxia-Induced Reactive Oxygen Species: Their Role in Cancer Resistance and Emerging Therapies to Overcome It

**DOI:** 10.3390/antiox14010094

**Published:** 2025-01-15

**Authors:** Eleicy Nathaly Mendoza, Maria Rosa Ciriolo, Fabio Ciccarone

**Affiliations:** Department of Biology, University of Rome Tor Vergata, 00133 Rome, Italy

**Keywords:** HIF, ROS, cancer resistance, antioxidants, tumor microenvironment

## Abstract

Normal tissues typically maintain partial oxygen pressure within a range of 3–10% oxygen, ensuring homeostasis through a well-regulated oxygen supply and responsive vascular network. However, in solid tumors, rapid growth often outpaces angiogenesis, creating a hypoxic microenvironment that fosters tumor progression, altered metabolism and resistance to therapy. Hypoxic tumor regions experience uneven oxygen distribution with severe hypoxia in the core due to poor vascularization and high metabolic oxygen consumption. Cancer cells adapt to these conditions through metabolic shifts, predominantly relying on glycolysis, and by upregulating antioxidant defenses to mitigate reactive oxygen species (ROS)-induced oxidative damage. Hypoxia-induced ROS, resulting from mitochondrial dysfunction and enzyme activation, exacerbates genomic instability, tumor aggressiveness, and therapy resistance. Overcoming hypoxia-induced ROS cancer resistance requires a multifaceted approach that targets various aspects of tumor biology. Emerging therapeutic strategies target hypoxia-induced resistance, focusing on hypoxia-inducible factors, ROS levels, and tumor microenvironment subpopulations. Combining innovative therapies with existing treatments holds promise for improving cancer outcomes and overcoming resistance mechanisms.

## 1. Introduction

Normal oxygen requirements are crucial for maintaining cellular homeostasis and ensuring proper metabolic functions. Adequate oxygen levels guarantee efficient ATP production in mitochondria, which supports energy-intensive processes and overall cell health. Hypoxia, or insufficient oxygen, disrupts these processes, leading to altered metabolism, increased oxidative stress, and impaired cellular functions, which can contribute to disease states such as cancer. Each tissue and organ have specific oxygen needs and supply rates. The variation in normal oxygen partial pressure (pO_2_) across different tissues and organs is due to their distinct characteristics, such as vascular density, types of blood vessels, cellular composition, extracellular matrix (ECM) components, and metabolic activity. Normal tissues typically maintain pO_2_ within a range of 23–70 mmHg (3–10% oxygen) thanks to a well-regulated oxygen supply mechanism and a responsive vascular network [1]. Hypoxic regions develop in about 90% of solid tumors due to several factors, leading to an environment that supports cancer progression and resistance to therapy. The tumor microenvironment (TME) is a continuously evolving entity formed by a complex collection of various types of cells, cancer cells, tumor stromal cells including fibroblasts, endothelial cells and immune cells and the components of ECM such as collagen, fibronectin, and others [2]. The quick and dysregulated expansion of tumors frequently surpasses the formation of new blood vessels, leading to a deficient and disorganized system that fails to deliver enough oxygen to all parts of the tumor [1]. This dysfunctional angiogenesis contributes to uneven oxygen distribution and areas of chronic hypoxia within the tumor. Tumor regions distant from blood vessels become increasingly hypoxic, while tumor areas near blood vessels are well oxygenated. As O₂ levels gradually decrease with distance, hypoxic necrotic regions often develop at the tumor core. Additionally, the high metabolic activity of cancer cells increases oxygen consumption, exacerbating hypoxic conditions, especially in the tumor core [1]. The lack of oxygen necessarily causes cancer cells to change their metabolism to rely more on glycolysis. Nevertheless, this oxidative-to-glycolytic metabolic shift also occurs in cancer cells regardless of pO_2_ variations, as first observed by Otto Warburg a century ago [3]. These adaptations allow cancer cells to thrive under fluctuating microenvironmental conditions that would be detrimental to normal cells, highlighting the complex interplay between metabolism and oxygen utilization in cancer. Furthermore, the increased interstitial pressure within tumors compresses blood vessels, impeding effective perfusion and oxygenation, which makes the penetration of drugs difficult [4]. The combination of these factors resulting in a hypoxic microenvironment also enhances tumor aggressiveness, metastasis, and resistance to traditional treatments.

During hypoxia, reactive oxygen species (ROS) are produced through several interconnected mechanisms, mainly mitochondrial dysfunctions and the activation of various pro-oxidant enzymes. Under low oxygen conditions, the electron transport chain in mitochondria becomes disrupted, as oxygen availability is insufficient to accept electrons. This disruption causes electron leakage, leading to the formation of superoxide [5]. In parallel, hypoxia-inducible factors (HIFs), which are the master regulators of the hypoxia response, also upregulate genes encoding ROS-generating enzymes such as NADPH oxidases (NOX; NOX1 and NOX4), which transfer electrons from NADPH to oxygen [3,6]. Additionally, xanthine oxidase activity can exacerbate ROS accumulation during hypoxia-reoxygenation and inflammatory responses triggered by low oxygen conditions [7].

**Figure 1:**
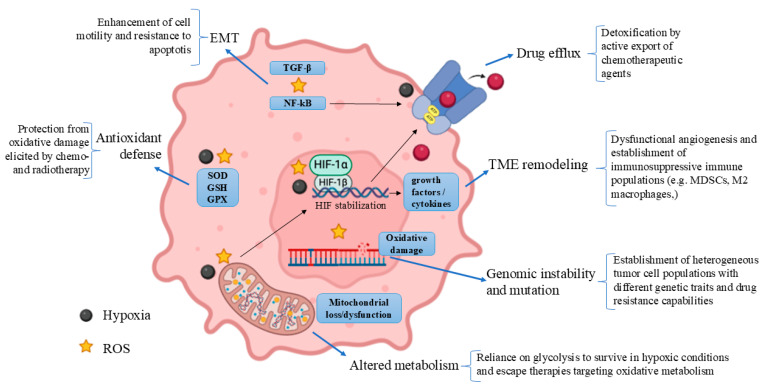
Mechanisms driven by hypoxia and ROS to induce cancer resistance. The hypoxic conditions determined by the TME and the associated ROS burst in cancer cells elicit different types of survival mechanisms that contribute to drug resistance. Oxidative stress-mediated DNA mutations and HIF-dependent changes in cell metabolism, drug efflux, and TME immunosuppression influence cellular adaptation and resistance to antitumor therapies, encouraging tumor aggressiveness and metastasis.

Hypoxia-induced ROS are highly associated with resistance to antitumor therapy [1]. The above-mentioned mechanisms involved in hypoxia-induced ROS production can contribute to oxidative stress and influence cellular adaptation and resistance by inducing genomic instability and mutations, changes in cell metabolism, modification of drug efflux, and adjustments in the TME, thus encouraging tumor aggressiveness and metastasis (Figure 1) [8,9]. ROS and hypoxia significantly influence the TME by promoting angiogenesis, immune suppression, and ECM remodeling, thereby enhancing tumor progression and therapy resistance. Hypoxia-induced ROS stabilize HIFs, driving processes such as disorganized angiogenesis and the recruitment of immunosuppressive cells, including tumor-associated macrophages (TAMs) and myeloid-derived suppressor cells (MDSCs) [10]. These immune cells, along with adaptive T and B cells, are reprogrammed in the TME to suppress antitumor immunity [11,12]. Additionally, ROS-mediated ECM remodeling creates physical barriers to immune cell infiltration and drug delivery, emphasizing the pivotal role of ROS in creating a tumor-supportive environment and highlighting the TME as a critical target for therapeutic intervention [13].

The different mechanisms that cancer cells use to survive and proliferate despite oxidative stress caused by hypoxia and ROS mainly rely on the stabilization of HIF transcription factors, which trigger survival pathways, support blood vessel growth, and increase glycolysis, redirecting metabolism from processes that depend on oxygen [3,8,14]. They also upregulate antioxidant defenses, such as glutathione (GSH) and superoxide dismutase (SOD), to neutralize ROS and mitigate oxidative damage [15,16]. These adaptive responses enable cancer cells to withstand hypoxia and ROS, contributing also to resistance against therapies by facilitating continued growth and survival.

Ongoing research and clinical trials are refining strategies to treat cancer and overcome drug resistance. Drugs such as HIF inhibitors, antioxidants, and TME modulators represent a diverse and promising class of therapies for overcoming hypoxia-induced ROS cancer resistance with continued research and trials to determine their potential and optimizing their use in cancer therapy. To effectively challenge this issue, a multifaceted approach targeting multiple aspects of tumor biology is essential. Integrating novel therapies with existing treatment modalities offers significant potential to enhance therapeutic outcomes and counteract resistance mechanisms. This review aims to explore the critical role of hypoxia-induced ROS in driving cancer resistance as well as to evaluate emerging therapeutic strategies that can be designed to overcome this challenge.

## 2. Hypoxia-Induced ROS and Cancer Resistance Development

### 2.1. Genomic Instability and Mutagenesis

ROS are highly reactive and can cause significant damages to cell structures, such as DNA. The genotoxic damage caused by ROS includes single- and double-strand breaks, base alteration as 8-oxoguanine, and crosslinks. If the damage is not repaired adequately, it may cause permanent mutations and increase the prevailing genomic instability, which provides a conducive atmosphere for the occurrence of additional mutational events. The loss of tumor suppressor genes, activation of oncogenes, and chromosome aberrations lead to tumor growth promotion. Moreover, genetic mutations result in the establishment of a heterogeneous tumor population, where cells in the same neoplasm have different genetic traits [17]. This genetic diversity is critical because it enables the selection of clones that are resistant to various therapies, including chemotherapy, radiation, and targeted therapies [18] (Figure 1). This adaptability poses a significant challenge, as even when a treatment is initially effective, resistant clones may survive, leading to relapse and disease progression.

Understanding the role of hypoxia-induced ROS in driving genomic instability and mutagenesis is crucial for developing more effective cancer therapies. Knowing the root causes of resistance, such as the hypoxic environment and the resulting ROS generation, could help minimize the chances of cancer relapse [17].

### 2.2. Cellular Adaptation and Survival

Hypoxia-induced ROS play a pivotal role in driving a wide range of cellular adaptations and survival mechanisms in cancer cells, which contribute to cancer resistance. The most relevant pathways activated by hypoxia-derived ROS are described hereafter.

#### 2.2.1. HIF Pathway Activation

An important role in hypoxia response is played by HIF proteins, which are commonly overexpressed in solid tumors. As already stated, the HIF pathway becomes active in low oxygen environments and plays a critical role in how cancer cells adapt to hypoxia [8,14]. HIFs are transcription factors composed of two components: an oxygen-sensitive alpha subunit (HIF-1α, HIF-2α, or HIF-3α) and a beta subunit (HIF-1β) that is always expressed. Under normal oxygen levels, HIF-α subunits are degraded via the ubiquitin–proteasome pathway following hydroxylation by prolyl hydroxylase (PHDs) enzymes. In contrast, under hypoxic conditions, PHD activity is limited by low oxygen tension and ROS accumulation, leading to HIF-1α stabilization [19]. When stabilized, HIF-1α moves to the nucleus, where it interacts with HIF-1β and activates the transcription of genes that support cell survival and adaptation, such as those regulating glycolytic metabolism (e.g., glucose transporters) and the angiogenic process, including vascular endothelial growth factor (VEGF) [8,19] (Figure 1). This activation allows cancer cells to thrive in hypoxic environments, making them also more resilient to therapies that depend on oxygen for efficacy.

#### 2.2.2. Promotion of Cell Survival Mechanisms

Hypoxia-induced ROS have a great impact in cellular signaling pathways that improve cancer cell survival and thus resistance to treatments. In low oxygen conditions, ROS can trigger the phosphoinositide 3-kinase/protein kinase B (PI3K/Akt) pathway, leading to cell survival, growth, and resistance to apoptosis through activation of downstream pro-survival and anti-apoptotic signals. The ROS-mediated stabilization of HIF-1α was also shown to activate the PI3K/Akt pathway, promoting cancer cell adaptation and survival in oxygen-deprived conditions [20]. Furthermore, hypoxia-induced ROS affect the mitogen-activated protein kinase (MAPK) pathway, which is also responsible for controlling cell growth, differentiation, and response to stress. This activation can result in increased cellular resistance to therapies that impinge on MAPK signaling, including both targeted and conventional treatments [21].

In parallel, hypoxia-induced ROS promote autophagy, enabling cancer cells to adjust and thrive by removing harmed organelles/proteins and providing necessary nutrients during periods of metabolic stress, leading to resistance to treatment and tumor progression. Autophagy also protects cells against damages caused by ROS preventing the accumulation of toxic cellular debris and avoiding cell death [22]. Hence, by modulating these pathways, ROS contribute to cancer cell survival and therapy resistance in the hypoxic TME [23].

#### 2.2.3. Activation of Defense Systems

Hypoxia-induced ROS improve the cancer cell detoxification mechanisms. One of the main detoxification systems involves glutathione S-transferases (GSTs), which induce the conjugation of anticancer drugs with GSH, forming water-soluble and less toxic compounds that are easier for the cell to excrete. Under hypoxic conditions, the enzymes responsible for GSH synthesis and GST activity are upregulated, which is partly due to HIF targeted transcription [24]. Furthermore, ROS lead to the activation of the nuclear factor erythroid 2-related factor 2 (Nrf2) pathway, further contributing to the upregulation of detoxification enzymes such as GSTs and NAD(P)H oxidoreductase 1 (NQO1). The Nrf2 pathway plays a central role in cellular antioxidant defense and is also very important in the development of cancer. Once released, Nrf2 translocates to the nucleus, where it binds to antioxidant response elements (AREs) and stimulates the expression of antioxidant genes [25]. The expression of a variety of antioxidant enzymes, including glutathione peroxidase, SOD and catalase, scavenges ROS and diminishes oxidative burst (Figure 1). Nrf2 activation not only suppresses oxidative damage but also supports cancer cells to escape apoptosis and to resist to oxidative stress elicited by chemo- and radiotherapy [26].

In parallel, the elevated activity of NOX and xanthine oxidase in cancer cells has been associated with increased tolerance to oxidative stress, promoting continuous cell growth and survival [27,28]. Xanthine oxidase, a key enzyme in purine catabolism, generates ROS during the oxidation of hypoxanthine to xanthine and xanthine to uric acid. These ROS act as signaling molecules, activating MAPK and PI3K/AKT pathways, which are crucial for cell proliferation, survival, and metastasis [29,30]. Under hypoxia, HIFs can also upregulate xanthine oxidase expression, enhancing ROS production and supporting cellular adaptation to oxygen deprivation [31]. Additionally, xanthine oxidase activity facilitates DNA damage repair mechanisms by modulating redox-sensitive enzymes and pathways, further enhancing cancer cell resistance to chemotherapy and radiotherapy [32].

#### 2.2.4. Induction of Epithelial-Mesenchymal Transition

Hypoxia and ROS are important in inducing epithelial–mesenchymal transition (EMT), which enhances the migration and invasion of cancer cells. ROS activate essential signaling pathways, including transforming growth factor-beta (TGF-β) and nuclear factor kappa-light-chain-enhancer of activated B cells (NF-κB), which are crucial for promoting EMT [33]. Recent studies indicate that ROS-induced TGF-β signaling increases the expression of mesenchymal markers and disrupts epithelial cell adhesion, thereby enhancing cell mobility and resistance to apoptosis [33] (Figure 1). Moreover, the activation of NF-κB by ROS also promotes EMT by increasing the levels of genes that play a role in cell survival and movement, which not only aid in metastasis but also in developing resistance to targeted therapies and chemotherapy [34,35].

### 2.3. Altered Metabolism

Under low oxygen conditions, the main metabolic alteration observed in cancer cells is the shift from oxidative phosphorylation to glycolysis. This shift occurs due to the stabilization of HIF-1α, which upregulates the expression of genes associated with glycolysis while repressing those involved in oxidative metabolism, such as the β-oxidation of fatty acids [36]. Additionally, ROS can activate AMP-activated protein kinase (AMPK), which is a key regulator of cellular energy balance that boosts glycolysis while inhibiting fatty acid synthesis, supporting cell survival during metabolic stress [37,38]. By glycolysis, cancer cells can produce ATP more quickly, albeit less efficiently, compared to oxidative phosphorylation; this metabolic shift allows cancer cells to thrive in hypoxic tumor environments by reducing their oxygen dependency [39]. Such metabolic adaptation not only supports rapid cell growth and proliferation but also helps cancer cells in evading therapies targeting oxidative metabolism by decreasing their dependence on oxygen [40] (Figure 1). By relying on glycolysis, cancer cells produce large amounts of lactate, which is exported by monocarboxylate transporters (MCTs), acidifying the TME [41]. This acidic environment supports cancer cell survival by facilitating invasion and metastasis, impairing immune cell function, and influencing pH-dependent enzymes and proteins involved in apoptosis [42]. In this way, cancer cells are able to evade immune detection and resist cell death.

Hypoxia and ROS-induced metabolic reprogramming also contribute to resistance against apoptosis during cancer therapies, the shift toward glycolysis allows cancer cells to maintain ATP production even when chemotherapy or radiation increases ROS levels [43]. This adaptation reduces mitochondrial oxidative stress, ensuring continuous energy production despite the oxidative damage caused by treatment. Moreover, the increased glycolytic activity under hypoxia promotes survival pathways, such as HIF-1α and AMPK, enhancing resistance to therapies [44]; consequently, cancer cells can survive and resist the apoptotic signals that typically arise from ROS accumulation during treatment.

### 2.4. Drug Efflux and Detoxification

Hypoxia and ROS can enhance cancer resistance by upregulating drug efflux pumps [45]. ATP-binding cassette (ABC) transporters actively expel chemotherapeutic agents from cancer cells, reducing their intracellular levels. During hypoxia, cancer cells often increase the expression of ABC transporters such as P-glycoprotein (P-gp/ABCB1), multidrug resistance-associated proteins (MRPs), and breast cancer resistance protein (BCRP/ABCG2) [46]. This upregulation is driven by stabilized HIFs that activate genes encoding these transporters [45]. As a result, cancer cells reduce the intracellular concentration of chemotherapeutic agents to sublethal levels, allowing them to survive and proliferate [47] (Figure 1).

Additionally, hypoxia-induced ROS can further enhance the activity of drug efflux pumps by modifying key signaling pathways. ROS activate NF-κB, which is also responsible for increasing ABC transporters [48]. This pathway amplifies both the expression and functionality of these pumps, improving drug-exporting capacity. The interplay of the HIF transcriptional network and ROS-induced signaling creates a robust mechanism that reduces drug accumulation in cancer cells, contributing to multidrug resistance (MDR) [45].

Furthermore, there is an interplay between drug efflux and detoxification mechanisms, which creates a synergistic defense system within cancer cells. After chemotherapeutic drugs are detoxified by GSH conjugation, the resulting drug conjugates often become substrates for ABC transporters, such as MRPs, which then actively transport these conjugates out of the cell [49]. This dual mechanism of detoxification followed by efflux significantly reduces the intracellular concentration of active drugs, further enhancing resistance. This system is particularly effective in maintaining the survival of cancer cells in the face of chemotherapeutic stress, making it a major contributor to treatment failure [50].

### 2.5. Modulation of the Tumor Microenvironment

ROS influence different processes in the TME, including angiogenesis, immune suppression, and ECM remodeling, thereby helping the tumor in evading treatment [51]. One key consequence of hypoxia-induced ROS is the stimulation of angiogenesis, which is the process of forming new blood vessels. In low-oxygen conditions, ROS stabilize HIFs, leading to an increased expression of VEGF [1]. VEGF facilitates the growth of new blood vessels, supplying tumors with oxygen/nutrients. Although these new vessels support tumor growth, they are often disorganized and leaky, resulting in persistent hypoxic regions within the tumor. This, in turn, sustains ROS production and alters the TME, ultimately making it harder for drugs to be delivered effectively, leading to resistance [51] (Figure 1).

ROS also impact the recruitment and polarization of TAMs and sustain their suppressive function via HIF and SIRT1-mediated regulation, contributing to immune evasion and resistance to cancer therapies. These macrophages are skewed toward an M2-like phenotype under the influence of ROS, promoting tumor progression and immunosuppression by secreting anti-inflammatory cytokines such as interleukin-10 (IL-10) and TGF-β [52].

Moreover, hypoxia-induced ROS significantly affect MDSCs, which is a heterogeneous population of immature myeloid cells that expand in the TME. Hypoxia changes mediated by HIF-1α and elevated ROS levels enhance the differentiation, recruitment, survival, and immunosuppressive functions of MDSCs, fostering a tumor-supportive microenvironment that promotes cancer progression, immune evasion, and therapy resistance. Hypoxic cancer cells secrete cytokines (CCL26, G-CSF, IL-6) to attract MDSCs to the TME, where HIF-1α drives the expression of ENTPD2, an enzyme that plays a key role in purinergic signaling within the TME, ensuring their survival and maintenance within the TME [10,53]. This supports their role in suppressing immune responses and promoting tumor progression, ensuring MDSC survival and maintenance. Through the secretion of molecules like arginase-1, nitric oxide, ROS, and TGF-β, MDSCs suppress T cell and NK cell activity, promote regulatory T cell expansion, and induce T cell exhaustion via programmed death-ligand 1 (PD-L1) expression, which then binds to the PD-1 receptor on T cells, leading to reduced T cell activity and immune evasion. Additionally, MDSC-derived exosomes carry suppressive factors that inhibit cytotoxic T lymphocyte activity [54,55]. By supporting angiogenesis and stromal remodeling, MDSCs further shield tumors from immune attacks and impede drug delivery, making them a significant target for improving therapies.

Additionally, ROS influence the functionality of adaptive immune cells, such as B and T cells. Hypoxia in the TME reprograms T cell metabolism, suppressing their effector functions and promoting immunosuppressive mechanisms that foster tumor growth, immune evasion, and therapy resistance. Lactic acid accumulation acidifies the extracellular space, impairing T cell activation, proliferation, and cytokine production [56]. Hypoxia further reduces key effector molecules like IFN-γ, TNF-α, and granzyme B in CD8^+^ cytotoxic and CD4^+^ helper T cells, weakening antitumor responses [57]. Additionally, it induces mitochondrial dysfunction and increases ROS, contributing to T cell exhaustion and resistance to PD-1/PD-L1 blockade therapies [58]. High levels of ROS in the TME impair T cell receptor (TCR) signaling, leading to reduced T cell activation and effector functions. Oxidative stress induced in T cells by ROS can trigger apoptosis and deplete the number of CTLs essential for antitumor immunity [11].

Furthermore, B cells in the TME exhibit reduced antibody production and impaired antigen presentation under oxidative stress conditions, which undermine their ability to mediate antitumor effects. Hypoxia interferes on B cell functions, while elevated HIF-1α activity disrupts early B cell development by impairing receptor expression and increasing pro-apoptotic factors, hindering antigen-specific differentiation [12]. Therefore, it also weakens long-term immunity by reducing germinal center formation and favoring low-affinity antibodies. Finally, regulatory B cells, expanded under hypoxia, secrete IL-10 and adenosine, suppressing inflammatory T cell activity and aiding immune evasion [59].

The remodeling of the ECM is another significant effect of hypoxia-induced ROS. ROS trigger matrix metalloproteinases (MMPs), which break down ECM elements and restructure it [60]. This remodeling process not only supports cancer cell invasion but also changes the mechanical characteristics of the TME, increasing stiffness and, therefore, creating a physical barrier around tumors that impedes immune cell infiltration and drug delivery and improves cancer cell viability and ability to resist cell death [13].

## 3. Therapeutic Strategy to Overcome Cancer Resistance Related to Hypoxia-Induced ROS

### 3.1. ROS Modulation

ROS include superoxide anion (O2^•−^), hydrogen peroxide (H_2_O_2_), and hydroxyl radicals (^•^OH). In cancer cells, ROS levels are often dysregulated, contributing to tumor development, progression, and resistance to treatments [61]. Cancer cells can exhibit both elevated ROS production and enhanced antioxidant defenses to survive oxidative stress. This dual characteristic enables them to utilize ROS for growth and survival while avoiding excessive ROS-induced damage that might cause cell death [62]. Considering that hypoxic conditions contribute to ROS production in cancer, strategies to overcome resistance and improve the effectiveness of cancer treatments by modulating ROS balance will be here presented. ROS modulators can be classified into ROS inducers and ROS scavengers (Table 1).

#### 3.1.1. ROS Inducers

Pro-oxidant therapies can overwhelm the antioxidant capacity of cancer cells by further increasing ROS levels, pushing oxidative stress beyond the threshold that can be tolerated and consequently leading to cell death. Some ROS inducers are able to increase ROS specifically in cancer cells [63]. Recent studies have explored the use of drugs like β-lapachone and Piperlongumine that selectively increase ROS levels in cancer cells, inducing apoptosis, while sparing normal cells [64,65]. Another example are the artemisinin derivatives, which are capable of inducing direct oxidative damage in cancer cells by the forming of ROS such as hydroxyl and superoxide anion radicals [66].

ROS inducers can be used alone or in combination with other therapies to overcome resistance. For example, combining ROS inducers with inhibitors of antioxidant systems can synergistically increase oxidative stress and promote cancer cell death [62]. Moreover, intensifying oxidative metabolism at the expense of glycolysis was shown to be effective in exacerbating the anticancer activity of ROS-promoting cytotoxic drugs [67,68].

#### 3.1.2. ROS Scavengers

Reducing ROS levels can also be beneficial, particularly in cancers where ROS-induced signaling contributes to survival and resistance. Antioxidants that reduce ROS can inhibit these pro-survival pathways.

- N-acetylcysteine (NAC): Known as a precursor of GSH and an efficient scavenger of hydroxyl radicals, NAC has been studied for its potential to decrease ROS levels, to inhibit cancer cell growth and overcome resistance when used in combination with other therapies [69]. It was confirmed that NAC could reverse gefitinib resistance in non-small cell lung cancer cells, also reversing EMT [70].
- Vitamin E is a lipid-soluble antioxidant that protects cell membranes from oxidative damage by scavenging free radicals, particularly peroxyl radicals, which are involved in lipid peroxidation. It has shown some potential in lowering ROS levels and overcoming resistance in specific cancer types [71]. Used as an adjuvant therapy, it protects normal cells during chemotherapy and radiotherapy, and it also reduces oxidative damage without interfering significantly with the therapeutic effects on cancer cells. In clinical studies, vitamin E has been explored for reducing the side effects of radiation therapy, particularly in head and neck cancers [72,73].
- Vitamin C is an antioxidant that scavenges ROS, reducing oxidative damage and making cancer cells more sensible to chemotherapy and radiotherapy, particularly in hypoxic tumors. Vitamin C can reduce HIF-1α activity, therefore reducing the expression of pro-angiogenic factors such as VEGF, restricting tumor growth [74].
- Bardoxolone-Methyl is a semi-synthetic triterpenoid that activates the Nrf2 pathway to increase ROS scavenging and reduce the side effects of cancer treatments by bolstering the antioxidant defenses [75]. Additionally, bardoxolone-methyl demonstrated to sensitize oral squamous cell carcinoma cells to radiotherapy, thus reducing tumor growth [76].
- Resveratrol is a natural polyphenol with antioxidant properties that scavenges ROS and modulates the activity of antioxidant enzymes, such as SOD and catalase [77]. It is able to reverse drug resistance in tumor cells by sensitizing them to chemotherapeutic agents. As an adjuvant chemotherapy agent, it enhances drug anticancer effects and decreases tumor volume by inhibiting cell proliferation and inducing apoptosis [78,79,80].
- Manganese porphyrin-based SOD mimics (SODm): These novel compounds mimic the properties of SOD enzymes, converting superoxide into H_2_O_2_ and O_2_ [81]. The most promising compounds of this group are MnTnHex-2-PyP5+ and MnTnBuOE-2-PyP5+ (BMX-001). These compounds are currently being explored for their ability to mitigate radiation-induced damage while also reducing cell viability in different types of cancer [82,83]. Additionally, these compounds have been shown to enhance the effectiveness of chemotherapy and radiotherapy. Thus, early-phase clinical trials are anticipated to further explore their therapeutic potential [84].

Challenges and future directions: ROS modulators are promising compounds to use in combination with chemotherapy, targeted therapy, or immunotherapy to enhance the antitumor response. Nevertheless, excessive ROS can damage normal tissues, leading to side effects, and it is thus important to study the therapeutic window of ROS modulators, as there is a fine line between therapeutic and toxic levels of ROS.

**Table 1:** In vitro, preclinical and clinical trial of ROS modulators as potential anticancer therapies.

Class	Compound	Type of Study	Cancer Type	Dose	Outcome	Ref.
ROS Inducer	β-Lapachone	preclinical	Liver	12.5 mg/kg/day for five days	Tumor growth inhibition	[64]
		in vivo (phase I)	Advanced solid tumors	MTD 390 mg/m^2^ every other week	Stable disease or tumor shrinkage	[85]
		in vivo (phase I)	Pancreatic	MTD 156 mg/m^2^ (+ gemcitabine and nab-paclitaxel)	Stable disease in 53%	[65]
	Piperlonguimine	in vitro	Thyroid	10.68 μM (24 h) 5.68 μM (48 h)	Growth inhibition, apoptosis, autophagy	[86]
		in vitro, preclinical		Single dose 10 mg/kg or 5 mg/kg	Good cytotoxic potential/ safety	[87]
ROS Scavenger	NAC	in vitro	NCSLC	NAC (5 mM) plus gefitinib (2 μM)	Restored gefitinib sensitivity	[70]
		clinical trial	Breast	Once a week I.V. (150 mg/kg) and twice daily orally (600 mg)	Reduced carcinoma cell proliferation.	[88]
	Vitamin E	in vivo (pilot)	Head and neck	100 IU (+500 mg vitamin C) + RT	Protects against RT effects	[72]
				Mouthwash with 0.2% vitamin E	Protects against RT effects	[73]
	Vitamin C	in vivo (pilot trial)	Pancreatic	50–100 g three times weekly + gemcitabine or erlotinib	7/9 patients had stable disease	[89]
		in vivo (pilot trial)	Ovarian	75–100 g twice weekly + Paclitaxel/carboplatin	Low chemotherapeutic toxicity, prolonged PFS	[90]
		preclinical	Ovarian, pancreatic	Twice daily 4 g/kg body weight	Decreased tumor growth	[91]
		in vitro	Melanoma	100 μM	Reduced progression	[74]
	Bardoxolone Methyl	in vitro	Oral squamous cell	10 nM associated with RT	Anti-cancer and radio-sensitizing effects	[76]
		in vivo (phase I)	Solid tumors, lymphomas	900 mg/day orally once daily	Prolonged stable disease of 4 or more months	[92]
	Resveratrol	in vitro	Breast	70 μM (+ sorafenib 6 μM)	Increased apoptosis	[78]
		in vivo (phase I)	Colorectal	5 g daily for 10–21 days	Increased apoptosis	[93]
	Manganese Porphyrin	in vitro	NCSLC	0.5 and 1 µM (alone or + cisplatin 1–5 µM)	Increased cell death and cisplatin cytotoxicity	[82]
		in vitro	Breast	5 µM (+doxorubicin 0.5–20 μM)	Reduced collective cell migration and chemotaxis	[81]
		in vitro	High-grade gliomas	28 mg loading + 14 mg maintenance dose for 2 times/week (+RT/temozolomide)	Promising early results on overall survival	[94]

MTD: maximum tolerated dose; RT: radiotherapy; I.V: intravenous; NCSLC: non-small cell lung cancer; PFS: progression-free survival.

### 3.2. HIF Inhibitors

Inhibitors of HIF signaling are emerging as promising therapeutic strategies to overcome cancer resistance induced by hypoxia and ROS (Table 2). These inhibitors aim to block the activity of HIFs, thereby disrupting the hypoxic response and making tumors more susceptible to treatment [95]. HIFs inhibitors can be classified into the following groups:

- Direct HIF inhibitors: These compounds directly target HIF-α subunits, preventing their stabilization under hypoxic conditions. Some examples are PT2385 and PT2399, inhibitors of HIF-2α, thus inhibiting the expression of its dependent genes such as VEGF-A and cyclin D1, showing tumor regression in preclinical models for clear cell renal cell carcinomas [96,97].
- Indirect HIF inhibitors: Indirect inhibitors may target upstream regulators of HIF-1α. For example, EZN-2208, a pegylated form of irinotecan, indirectly inhibits HIF-1α by decreasing its transcriptional activity. The results of clinical trials showed a reduced tumor growth in patients with advanced solid tumors, and at the same time, it was well tolerated [98,99]. Other examples are the histone deacetylase inhibitors, such as vorinostat and romidepsin, which are novel drugs that decrease HIF-1α stabilization by inducing its degradation [100,101].

Combination therapies with HIF inhibitors are also being tested. Combining HIF inhibitors with chemotherapeutic agents such as doxorubicin demonstrated a synergistic cytotoxic effect, making tumors more susceptible to treatment [102]. Moreover, as hypoxia also makes tumor cells less responsive to radiotherapy, the use of HIF inhibitors can decrease the cellular defense against ROS, thereby increasing DNA damage in cancer cells and enhancing the effectiveness of radiation therapy [103].

Challenges and future directions: One major issue is the potential toxicity, since HIFs also have vital functions in normal tissues. Developing inhibitors that specifically target the TME is essential to reduce the occurrence of off-target effects. Moreover, tumors can trigger alternative mechanisms that circumvent HIF inhibition, resulting in resistance to treatment similar to other targeted therapies. It is important to understand the reasons behind this resistance and develop strategies to overcome it, such as using combination therapies or sequential treatment regimens. The efficacy of HIF inhibitors may differ on the level of hypoxia and the type of tumor. Taking into consideration this diversity, customized treatment approaches are expected to be more successful.

### 3.3. Metabolic Pathways Inhibitors

Targeting metabolic pathways has emerged as a potential therapeutic strategy (Table 2), given the role of altered metabolism in promoting cancer resistance. Inhibitors of glycolysis and inhibitors of lactate production or transport (such as MCT inhibitors) are being explored [41,104].

- Glycolysis modulators: Deoxyglucose (2-DG) was studied in preclinical trials as an adjuvant therapy with chemotherapy and demonstrated sensitization to cell death and inhibition of tumor growth. Additionally, in early-phase clinical trials, it was observed tolerable and with reversible adverse effects [105]. PFKFBs (phosphofructo-2-kinase/fructose-2,6-bisphosphatase) are enzymes that regulate the transformation between fructose-2, 6-bisphosphate, and fructose-6-phosphate in glucose metabolism. PFKFB3 acts as a vital regulator of glycolysis that promotes cancer cell proliferation, migration, survival, metastasis, and resistance [106]. It catalyzes the production of fructose-2,6-bisphosphate, a potent allosteric activator of phosphofructokinase 1 (PFK-1); therefore, it is involved in the enhancement of glycolytic activity [107]. PFKFB3 inhibitors, such as PFK-158, reduce glucose consumption and lactate production, impairing energy supply and growth, thus showing antitumor activity in preclinical studies for melanoma, breast, and lung cancers [108].
- Glutaminolysis inhibitors: CB-839 (telaglenastat) shows anti-proliferative activity in solid tumor models (breast cancer, non-small cell lung cancer, renal cell carcinoma) both alone and in combination with other anti-cancer therapies in preclinical studies [109].
- Lipid metabolism inhibitors: Lipids are crucial for various cellular functions, including membrane synthesis, energy storage, and signal transduction [110]. Targeting lipid metabolism pathways has emerged as a promising strategy in cancer treatment. Opaganib and TVB-2640 are some examples still under clinical trials [111,112].
- Inhibition of mitochondrial metabolism: Mitochondrial bioenergetics and signaling are required for cancer initiation and survival [113]. Therefore, researchers have begun to study the development of new antineoplastic agents that target the mitochondria [113]. Devimistat is a novel drug designed to inhibit mitochondrial metabolism. It specifically targets enzymes within the Krebs cycle, such as the inactivation of PDH and KGDH, which are crucial for energy production in cells [114,115].

Challenges and future directions: Metabolic inhibitors can also affect normal cells, leading to off-target effects and toxicity in normal cells. Developing strategies to selectively target cancer cells while protecting normal tissues is still a major difficulty. Metabolic plasticity is the term given to the switch between different metabolic pathways to avoid the effects of targeted inhibitors, therefore causing cell populations to use different metabolic pathways to sustain their survival and growth, ultimately affecting their response to therapies. In consequence, achieving sustained therapeutic responses with single-agent metabolic inhibitors is difficult due to their adaptability. Therefore, the future of metabolic inhibitors likely relies on their association with chemotherapeutic or immunotherapeutic agents. Understanding the complexity between the cancer metabolic pathways and the metabolic interactions will be key for developing effective therapeutic strategies.

**Table 2:** Preclinical, clinical and ongoing clinical trials of HIF inhibitors and metabolic inhibitors in cancer.

Class	Compound	Type of Study	Cancer Type	Dose	Outcome	Ref.
HIF inhibitor	PT 2385	preclinical	Clear cell renal cell	30 or 100 mg/kg twice daily	Tumor regressions in mouse model	[97]
		in vivo (phase I)	Clear cell renal cell	100 to 1800 mg twice daily	Favorable safety profile and promising efficacy	[116]
	PT 2399	preclinical	Clear cell renal cell	30 mg/kg twice daily	Tumor regression in mouse model	[96]
	EZN-2208	preclinical	Glioblastoma	Single I.V. injection 30 mg/kg	Direct effects on the tumor vasculature	[98]
		in vivo (phase I)	Advanced solid	I.V. 1.25 mg/m^2^ and 25 mg/m^2^ once every 21 days	Well-tolerated and stable disease in 41% of the patients	[99]
		in vivo (phase I)	Refractory solid	I.V. once every 21 days (12–30 mg/m^2^)	Well-tolerated and associated with clinical benefit in patients with neuroblastoma.	[117]
	Vorinostat	in vivo (phase I/II)	Renal cell carcinoma	Orally, 200 mg/twice a day + bevacizumab	Associated with clinical benefit	[100]
	Romidepsin	in vivo (phase II)	Carcinoma of the head and neck	Infusion 13 mg/m^2^ on days 1, 8 and 15 of 28 days cycles	As a single agent has limited activity	[101]
			Lymphoma	Infusion 14 mg/m^2^ on days 8, 15, 22 of a 35-day cycle	Ongoing	[118]
Metabolic inhibitors	2-DG	in vivo (phase I)	Advanced solid	Orally, once daily (2 mg/kg) + docetaxel	32% of patients had stable disease, 3% had partial response	[105]
	CB-839	in vivo (phase II)	Renal cell carcinoma	Orally, 800 mg twice daily + everolimus	Well-tolerated and improved progression-free survival	[109]
		in vivo (phase II)	Advanced Cervical	Orally, 800 mg twice per day + Chemoradiation	Ongoing	[119]
	TVB-2640	in vivo (phase II)	Glioblastoma	100 mg/m^2^ daily + bevacizumab 10 mg/kg	Well-tolerated and improved overall response rate	[112]
				100 mg/m^2^ daily + bevacizumab 10 mg/kg	Ongoing	[120]
		in vivo (phase I)	Prostate	100 mg + Enzalutamide 160 mg once daily for 28 days	Ongoing	[121]
	Opaganib	in vivo (phase II)	Prostate	Orally 250/500 mg twice a day + (Abiraterone/enzalutamide)	Ongoing	[111]
	Devimistat	In vivo (phase I)	Biliary Tract	Infusion of 2000 mg/m^2^ + gemcitabine and cisplatin	Well-tolerated with an acceptable safety profile	[115]
		In vivo (phase I)		250 mg/m^2^; 500 mg/m^2^; 1000 mg/m^2^	Ongoing	[122]

I.V: intravenous; PDAC: pancreatic adenocarcinoma.

### 3.4. Modulating TME

TME regulates tumor cell survival and function; therefore, modulating the TME seems to be a promising therapeutic strategy to overcome hypoxia-induced resistance. Some strategies include anti-angiogenic therapies with aim to normalize tumor vasculature, targeting metabolic shift, reprogramming the immune microenvironment, and improving oxygenation in the tumor [123].

- Normalizing tumor vasculature: Strategies to normalize the atypical tumor vasculature can enhance oxygen delivery to the tumor, thereby reducing hypoxia and improving the efficacy of therapies. Agents such as bevacizumab, aflibercept, and ramucirumab are being studied for their potential to normalize blood vessels and reduce hypoxia-induced resistance by targeting VEGF signaling [124,125].
- Reprogramming the immune microenvironment: therapies targeting tumor-associated macrophages to shift them from a pro-tumorigenic and pro-angiogenic M2 phenotype to an antitumor M1 phenotype using Toll-like receptor agonists with cytokines or anti-CD47 are under research [126].
- Oxygen delivery systems: Innovative approaches to deliver oxygen directly to attenuate tumor hypoxia and enhance cancer therapy are being studied. Oxygen-carrying nanoparticles, hyperbaric oxygen therapy, and oxy-mimetics are some of the strategies in research to increase oxygen levels within the TME. Thus, oxygen delivery systems to improve cancer treatment hold great potential for future clinical translation [127].

Challenges and future directions: The high heterogeneity of the TME with variations in oxygen levels, cell populations, and metabolic states complicates the development of universal therapeutic strategies. Additionally, combination therapies are essential for overcoming possible resistance to the therapeutic strategies, as cancer cells and the TME can adapt.

## 4. Conclusions

Hypoxia-induced ROS play a crucial role in promoting cancer progression, resistance against therapies, and metastasis by modifying tumor cell metabolism, increasing genomic instability, and establishing a favorable environment for the growth and survival of cancer cells. The main difference between the impact of hypoxia-induced ROS and the ones coming from other sources (e.g., chemotherapy itself) is the leading contribution of TME derangement that elicits pro-oxidant conditions accompanied by an HIF-mediated adaptive response. These complex interactions represent a challenge for effectively treating cancer; advances in understanding the molecular mechanisms behind hypoxia and ROS have led to the development of promising therapeutic strategies aimed at reducing their effects. Emerging therapies such as HIF inhibitors, ROS modulators, metabolic pathways inhibitors and TME modulators could be useful to enhance the efficacy of existing cancer therapies and to overcoming resistance.

Targeting the TME and inhibiting HIF currently stand out as the most promising strategies for overcoming cancer resistance. Approaches involving TME modulators aim to adjust tumor vasculature, target metabolic shift, improve oxygenation in the tumor and reprogram the immune landscape of tumors, reducing their immunosuppressive nature and enhancing their responsiveness to immune checkpoint inhibitors. In parallel, targeting the HIF pathway is gaining significant attention, as hypoxia and HIF are critical in cancer progression and resistance, particularly by promoting angiogenesis. These multifaceted therapeutic strategies disrupt such processes, increasing the tumor’s sensitivity to therapies, especially when combined with chemotherapy or radiotherapy. However, further research is needed to optimize their use for overcoming hypoxia-induced resistance and to ensure efficacy and safety.
